# The lived experience of frailty: beyond classification and towards a holistic understanding of health

**DOI:** 10.1007/s41999-023-00909-4

**Published:** 2024-01-09

**Authors:** Chenhui Chenhuichen, Aisling M. O’Halloran, Deirdre Lang, Rose Anne Kenny, Roman Romero-Ortuno

**Affiliations:** 1 Geriatrics Department, Hospital Universitario de Navarra, Pamplona, Navarra Spain; 2 The Irish Longitudinal Study on Ageing (TILDA), Trinity College, Dublin, Ireland; 3 Discipline of Medical Gerontology, 6th Floor, School of Medicine, Trinity College, Mercer’s Institute for Successful Ageing, St James’s Hospital, Dublin 8, Ireland; 4 National Frailty Education Programme, Office of the Nursing & Midwifery Service Director (ONMSD), Health Service Executive, Dublin, Ireland; 5 Global Brain Health Institute, Trinity College Dublin, Dublin, Ireland

**Keywords:** Frailty, Lived experience, Ageing perceptions, Psychology, Ageism, Comparative study

## Abstract

**Purpose:**

Frailty is characterised by decreased physiological reserves and vulnerability to stressors. Although scales, such as the Fried’s Frailty Phenotype (FP), Frailty Index (FI), and Clinical Frailty Scale (CFS), are used to identify frailty, the lived experience of frailty remains understudied.

**Methods:**

This cross-sectional observational research involved participants aged 65 years and older from Wave 1 of The Irish Longitudinal Study on Ageing (TILDA). Participants were categorised into four independent groups: three frail groups based on the aforementioned scales and a non-frail group. Quantitative variables, including self-rated health, CASP-19 quality-of-life score, and frequency of social activities, were analysed and described.

**Results:**

The study encompassed 1999 participants with an average age of 72 years, of whom 51% were women. FP exclusively identified 1.6% as frail (*n* = 32), FI 11.7% (*n* = 233), and CFS 6.8% (*n* = 135). More than 60% of all those classified as frail reported their health as good, very good, or excellent, with the lowest proportion (64%) being among frail by FI participants. Frail by FI participants exhibited the lowest mean average CASP-19 score, yet it remained relatively high at 39 out of 57 points. Over 77% of all frail individuals engaged in active leisure activities at least once a month.

**Conclusion:**

This study underscores the need to comprehend frailty holistically beyond its mere identification. It challenges the prevailing belief that frailty inevitably leads to impaired quality of life and limited social engagement. The findings advocate for a reassessment of how both the general public and healthcare professionals perceive frailty.

## Key summary points

**Aim:**

We cross-sectionally described measured characteristics and lived experiences of older adults classified as frail by three different scales in The Irish Longitudinal Study on Ageing (TILDA).

**Findings:**

Despite being classified as frail, more than 60% rated their health positively, and over 77% engaged in active leisure activities at least once a month.

**Message:**

This study challenges the excessively negative perceptions of frailty, underscoring the need for a holistic understanding of frailty beyond classification scales.

## Introduction

Frailty is characterised by impaired functioning and reduced physiological reserves, and it is defined as a medical syndrome or state that increases an individual’s vulnerability for developing increased dependency and/or mortality when exposed to a stressor [1, 2]. In recent years, the concept of frailty and its classification tools have been used to evaluate outcomes in various clinical settings, such as oncology [3], surgery [4], intensive care medicine [5] or drug efficacy [6]. Although the medical concept of frailty is clear and many studies have identified it as a risk factor for adverse health outcomes including falls, hospitalisations and mortality [7, 8], there are many scales that can be used to identify frailty in older adults. Frequently used tools include the physical frailty phenotype (FP) [9], the frailty index (FI) of cumulative deficits [10, 11], and the clinical frailty scale (CFS) [12, 13].

Some scholars have evidenced the considerable heterogeneity among older people living with frailty when the identification has been performed with different tools. For example, Xue et al. [14] concluded that the FI and FP could not be interchangeable, as only 12% of their sample were identified as frail by both scales in their study. Additionally, Romero-Ortuno et al. [15] demonstrated that in Irish community-dwelling older adults, different frailty scales had different ability to predict 8-year mortality.

Moreover, most frailty scales usually assess physical function and/or comorbidities, thus excluding other essential health-related aspects, such as mood, quality of life, or social factors. According to the Oxford Dictionary, the term "lived experience" refers to the "personal knowledge about the world gained through direct, first-hand involvement in everyday events rather than through representations constructed by other people" [16]. Although, on average, older people living with frailty tend to have a lower quality of life than those classified as non-frail [17], Crowder et al. [18] warned against generalisations when they reported that five days after receiving chemotherapy, frail older people with cancer actually reported a better quality of life than those who were classified as robust.

While understanding the lived experience of older people living with frailty can be highly valuable in designing improvements in their hospital care pathways [19], there is limited evidence evaluating the lived experience of frailty as identified by different tools in the community setting. Therefore, the aim of our study was to cross-sectionally evaluate the lived experience of older people classified as frail by different scales in The Irish Longitudinal Study on Ageing (TILDA).

## Methods

### Design and setting

Participants from Wave 1 of TILDA were included in the present study. TILDA is a population-based longitudinal study that collects information on the mental and physical health, economic and social circumstances of people aged 50 and over in Ireland. The assessment was conducted through a home interview, a self-completion questionnaire, and, in some participants, a detailed health centre assessment. The methodology of the study has been extensively described elsewhere [20–22].

### Participants

This was a secondary analysis of the dataset that formed the basis of the previously published study by Romero-Ortuno et al. [15], which investigated the ability of different frailty scales to predict 8-year mortality in TILDA. For the current study, our focus was on Wave 1 participants aged 65 years or older who had complete data for frail state classification according to the available frailty tools. The age cut-off of 65 years and over was chosen to align with the age group where frailty tools are typically recommended for use [23].

### Measures

The four available frailty measures in TILDA were FP, FI, CFS, and FRAIL [24, 25]. As illustrated in Fig. 2 of the previously published paper [15], participants were divided into mutually exclusive groups based on these scales (32 frail participants solely by FP, 233 by FI, 135 by CFS, and 3 by FRAIL), with one group consisting of 1599 non-frail participants. Due to the quantitative nature of the present study, it was decided not to include the 3 participants identified as frail by FRAIL in the analysis.

Briefly, participants were considered frail by FP if they had ≥ 3 features (exhaustion, unexplained weight loss, weakness, slowness, and low physical activity). The operationalisation of the frailty phenotype in TILDA followed the same criteria as in the Cardiovascular Health Study [9], with the exception of the physical activity criterion, for which we used the short form of the International Physical Activity Questionnaire [26]. As described elsewhere, participants were classified as frail based on a 32-item FI (FI ≥ 0.25) [27] and the CFS classification tree (CFS ≥ 5) [28].

For group characterisation purposes, “measured” participant characteristics included sociodemographics (age, sex, and proportions of third-level education, low socio-economic group [manual skilled, semi-skilled or unskilled], living alone, and rural provenance); body mass index (BMI, kg/m^2^); number of self-reported chronic conditions (counted from the following list: heart attack or heart failure or angina, cataracts, hypertension, high cholesterol, stroke, diabetes, lung disease, asthma, arthritis, osteoporosis, cancer, Parkinson’s disease, peptic ulcer, and hip fracture); proportion of having a long-term illness self-described as “limiting”; number of regular medications excluding supplements; polypharmacy defined as being on 5 or more regular medications; number of self-reported difficulties in basic activities of daily living (BADL, counted from the following list: dressing, including putting on shoes and socks; walk across a room; bathing or showering; eating, such as cutting up food; getting in or out of bed; and using the toilet, including getting up or down); number of self-reported difficulties with independent activities of daily living (IADL, counted from this list: preparing a hot meal; doing household chores e.g., laundry, cleaning; shopping for groceries; making telephone calls; taking medications; and managing money such as paying bills and keeping track of expenses); having at least 1 fall in the past year; and the Mini-Mental State Examination (MMSE) score [29].

To evaluate the lived experiences of the participants, we collected the following quantitative information:

- Abbreviated Penn State Worry Questionnaire [30] (scores ranging between 0 and 40), with higher scores indicating higher levels of worry.
- Being afraid of falling (yes vs. no).
- Center for Epidemiologic Studies Depression Scale (CES-D) (0–60), with a cut-off value of ≥ 16 identifying significant depressive symptomatology [31].
- Hospital Anxiety and Depression Scale-Anxiety (HADS-A) (0–21), with a cut-off score of ≥ 8 for anxiety screening [32].
- The quality-of-lifeCASP-19 scale 28 (0–57), with higher scores indicating greater well-being. The CASP-19 includes 6 items for control, 5 for autonomy, 4 for pleasure and 4 for self-realisation [33].
- A 5-item version of the UCLA loneliness scale (0–10), with higher scores indicating greater levels of loneliness [34].
- Proportions of having at least two close friends or relatives (social ties); at least one intimate social relationship; at least one formal organisational involvement; partaking in any active or social leisure at least once a month (i.e., go out to films, plays and concerts; attend classes and lectures; travel for pleasure; play cards, bingo, games in general; go to the pub; eat out of the house; participate in sport activities or exercise); partaking in any passive or solitary leisure activity at least once a month (i.e., watch television; work in the garden, or your home, or on a car; read books or magazines for pleasure; listen to music, radio; spend time on hobbies or creative activities).
- The 32-item Ageing Perceptions Questionnaire (APQ) assesses self-perceptions of ageing along the following domains: acute/chronic timeline (5 items, with higher scores indicating constant higher awareness of one’s ageing), cyclical timeline (5 items, with higher scores indicating higher intermittent awareness of one’s ageing), positive consequences (3 items, with higher scores indicating more perceived growth and wisdom as a consequence of one’s ageing), negative consequences (5 items, with higher scores indicating more perceived problems and restrictions as a result of one’s ageing), positive control (5 items, with higher scores indicating a more internal locus of control), negative control (4 items, with lower scores indicating a more external locus of control), and emotional representations (5 items, with higher scores indicating greater worry and uncertainty about the future) [35]. The mean score for each subscale (minimum: 1; maximum: 5) is calculated.
- Proportion of self-rated memory and overall health (relative to others of same age) being good, very good, or excellent.

### Statistical analyses

All statistical analyses were carried out using IBM SPSS Statistics for Windows (version 26.0. Armonk, NY: IBM Corp). Data were presented as mean with standard deviation (SD) or median with interquartile range (IQR) for continuous variables, or count with percentage (%) for categorical variables. To compare a dichotomous variable across groups, we employed the Chi-square test; and to test if the distribution of a continuous variable was the same across groups, we employed the 2-sided independent-samples Kruskal–Wallis test. When an overall difference was identified, we conducted pairwise comparisons and reported the ones that were statistically significant. We adjusted the level of statistical significance at *p* ≤ 0.001 as per Bonferroni correction for multiple tests.

### Ethics

The study was conducted according to the guidelines of the Declaration of Helsinki, and approved by the Faculty of Health Sciences Research Ethics Committee at Trinity College Dublin, Dublin, Ireland (wave 1, reference: ‘The Irish Longitudinal Study on Ageing’, date of approval: May 2008). Written informed consent was obtained from all participants included in the study.

## Results

A total of 1999 participants were included in the present study, with a mean age of 72.0 years (SD 5.7, range 65–94), and 1021 (51.1%) being female.

Table 1 compares the measured characteristics of the four mutually exclusive groups (1599 non-frail, 32 frail solely by FP, 233 by FI, and 135 by CFS). All three frail groups were significantly older than the non-frail group (*p* ≤ 0.001). Although the FP group was on average 3 years older than the other two frail groups, this did not reach statistical significance. The FI group had a higher proportion of females (66%) compared to the other groups. Additionally, the FP group had the lowest BMI. In terms of multimorbidity, the FI group had a higher number of chronic conditions (median of 4 vs. 2 for the other groups), a higher proportion of limiting long-term illness (54%), and a higher number of regular medications (median of 6) and polypharmacy (72%). The CFS group demonstrated a concentration of both BADL and IADL disability. The three frail groups experienced more falls in the past year compared to the non-frail group, with the highest proportion of fallers in the FP group (38%). Furthermore, the FP group had the lowest mean MMSE score (25 points).

**Table 1:** Measured participant characteristics

	NF (*n* = 1599)	FP (*n* = 32)	FI (*n* = 233)	CFS (*n* = 135)	Overall* p* value and significant pairwise comparisons
Mean age (SD), years	71.5 (5.5)	77.0 (5.9)	73.6 (5.9)	74.1 (6.5)	< 0.001* NF-FP NF-FI NF-CFS
Female sex (%)	49.2	34.4	66.1	51.1	**< 0.001**^**#**^ FI-NF FI-FP FI-CFS
Third-level education (%)	30.0	16.1	21.2	26.2	0.018^#^
Socio-economic group: manual skilled, semi-skilled or unskilled (%)	24.3	19.2	28.3	26.9	0.576^#^
Lives alone (%)	26.3	31.3	34.3	31.9	0.046^#^
Rural provenance (%)	43.3	52.2	43.7	37.8	0.582^#^
Mean BMI (SD), Kg/m^2^	27.8 (0.1)	26.1 (1.3)	27.9 (0.6)	27.9 (0.7)	0.001* FI-FP FI-NF
Median number of chronic conditions (IQR)	2.0 (2.0)	2.0 (2.0)	4.0 (2.0)	2.0 (2.0)	< 0.001* FI-NF FI-FP FI-CFS
Limiting long-term illness (%)	13.0	34.4	53.6	36.3	**< 0.001**^**#**^ NF-FP NF-FI NF-CFS FI-NF FI-FP FI-CFS
Median number of medications (IQR)	2.0 (3.0)	4.0 (3.0)	6.0 (4.0)	4.0 (3.0)	< 0.001* NF-FP NF-FI NF-CFS FI-FP FI-CFS
Polypharmacy (%)	20.7	44.8	72.3	35.3	**< 0.001**^**#**^ NF-FP NF-FI NF-CFS FI-FP FI-CFS
Any BADL disability (%)	0.0	0.0	0.0	74.8	**< 0.001**^**#**^ CFS-NF CFS-FP CFS-FI
Any IADL disability (%)	0.0	0.0	0.0	35.6	**< 0.001**^**#**^ CFS-NF CFS-FP CFS-FI
At least 1fall in the last year (%)	19.3	37.5	28.9	26.7	**< 0.001**^**#**^ NF-FP NF-FI NF-CFS
Mean MMSE score (SD)	27.7 (3.3)	25.1 (5.3)	26.1 (6.2)	26.5 (5.1)	< 0.001* NF-FP NF-FI

*NF* non-frail, *FP* frailty phenotype, *FI* frailty index, *CFS* clinical frailty scale, *BMI* Body Mass Index, *MMSE* Mini-Mental State Examination, *BADL* basic activities of daily living, *IADL* independent activities of daily living, *SD* standard deviation, *IQR* interquartile range

*Independent-samples Kruskal–Wallis test

^#^Chi-square test

Significant differences are highlighted in bold

Regarding the lived experiences, Table 2 displays the mean scores of the evaluated scales across the different groups. In general, worry levels were low in the frailty groups, ranging from a mean of 13–17 points out of 40 in the abbreviated Penn State Worry Questionnaire, and there were no significant differences compared to the non-frail group. Frail individuals exhibited a higher prevalence of fear of falling compared to their non-frail counterparts. Depressive symptoms were very low across frail groups, ranging from a mean of 5 to 7 points out of 60 in the CES-D scale, compared to a mean of 4 points in the non-frail group. Anxiety symptoms were also generally low (mean HADS-A of 5 to 6 points across frailty groups, compared to 4 points in the non-frail group). In terms of quality of life, the FI group had the lowest mean of 39 out of 57 points in the CASP-19 scale, followed by 41 points in the FP group, 44 points in the CFS group, and 46 points in the non-frail group. In terms of quality-of-life subdimensions, although frail groups had lower scores in autonomy, control, and self-realisation, their pleasure scores were not different even in comparison with the non-frail group (14 points across the board). Mean loneliness was higher in the FI group, but the average severity was low with a mean score of 3 out of 10, compared to a mean of 2 in the other three groups.

**Table 2:** Lived experience characteristics

	NF (*n* = 1599)	FP (*n* = 32)	FI (*n* = 233)	CFS (*n* = 135)	Overall* p* value and significant pairwise comparisons
Worry: mean aPSWQ score (SD) [0–40]	13.8 (0.2)	16.6 (1.7)	14.3 (0.6)	13.4 (0.6)	0.117*
Afraid of falling (%)	21.7	53.1	38.6	37.8	**< 0.001**^**#**^ NF-FP NF-FI NF-CFS
Depression: mean CES-D score (SD) [0–60]	4.1 (0.1)	6.8 (1.1)	6.8 (0.5)	5.4 (0.5)	< 0.001* NF-FI
Anxiety: mean HADS-A score (SD) [0–21]	4.4 (0.1)	5.9 (0.8)	5.4 (0.3)	4.5 (0.3)	0.004*
Quality of life: mean CASP-19 score (SD) [0–57]	46.0 (0.2)	41.1 (1.9)	39.4 (0.9)	43.8 (0.9)	< 0.001* FI-NF FI-CFS
Autonomy	11.7 (0.1)	10.6 (0.6)	10.4 (0.2)	11.3 (0.3)	< 0.001* FI-NF FI-CFS
Control	9.0 (0.1)	7.7 (0.6)	7.5 (0.2)	8.4 (0.2)	< 0.001* FI-NF FI-CFS
Pleasure	14.2 (0.0)	14.1 (0.3)	13.7 (0.1)	13.9 (0.2)	0.003*
Self-realisation	11.6 (0.1)	10.3 (0.4)	10.3 (0.2)	10.9 (0.3)	< 0.001* FI-NF
Loneliness: mean UCLA score (SD) [0–10]	1.7 (0.1)	2.3 (0.5)	2.5 (0.2)	1.7 (0.2)	< 0.001* FI-NF
≥ 2 close social ties (%)	98.9	96.8	98.2	98.5	0.620^#^
Intimate relationship (%)	51.1	46.2	57.8	56.5	0.199^#^
Formal organisational involvement (%)	72.9	44.4	61.4	69.4	**< 0.001**^**#**^ NF-FP NF-FI
Partakes in active leisure (%)	90.1	77.8	82.1	85.5	**0.001**^**#**^ NF-FI
Partakes in solitary leisure (%)	98.8	96.3	98.1	100	0.291^#^
Ageing Perceptions Questionnaire (APQ)					
Mean acute/chronic timeline score (SD)	2.6 (0.8)	3.2 (1.0)	3.0 (0.9)	2.9 (0.9)	< 0.001* NF-FP NF-FI
Mean cyclical timeline score (SD)	2.7 (0.8)	3.2 (0.8)	3.0 (0.8)	2.8 (0.8)	< 0.001* NF-FI
Mean positive consequences score (SD)	3.7 (0.7)	3.9 (0.8)	3.7 (0.8)	3.8 (0.6)	0.322*
Mean negative consequences score (SD)	2.9 (0.8)	3.6 (0.7)	3.4 (0.7)	3.2 (0.8)	< 0.001* NF-FP NF-FI NF-CFS
Mean positive control score (SD)	3.9 (0.6)	3.9 (0.7)	3.8 (0.7)	4.0 (0.5)	0.006*
Mean negative control score (SD)	3.1 (0.8)	2.6 (0.8)	2.9 (0.7)	2.9 (0.9)	< 0.001* FI-NF
Mean emotional representations score (SD)	2.2 (0.7)	2.6 (0.9)	2.5 (0.8)	2.3 (0.9)	0.002*
Self-rated health (relative to others of same age): good, very good or excellent (%)	93.2	71.9	63.9	85.2	**< 0.001**^**#**^ NF-FP NF-FI NF-CFS FI-CFS
Self-rated memory: good, very good or excellent (%)	85.5	71.9	67.0	81.5	**< 0.001**^**#**^ FI-NF FI-CFS

*NF* non-frail, *FP* frailty phenotype, *FI* frailty index, *CFS* clinical frailty scale, *aPSWQ* abbreviated Penn State Worry Questionnaire, *SD* standard deviation, *CES-D* Center for Epidemiologic Studies Depression Scale, *HADS-A* Hospital Anxiety and Depression Scale-Anxiety, *APQ* Ageing Perceptions Questionnaire

*Independent-samples Kruskal–Wallis test

^#^Chi-square test

Significant differences are highlighted in bold

In terms of social variables, the FP group reported the lowest proportion of formal organisational involvement (44% compared to 73% in the non-frail group) and engagement in active leisure activities, but the latter proportion was still a remarkable 78% compared to 90% in the non-frail group. There were no statistically significant differences in other social variables across the board, with proportions of close social ties ranging from 97 to 99%, engagement in solitary leisure activities ranging from 96 to 100%, and having an intimate relationship ranging from 46 to 58%.

Perceptions of ageing were found to differ across groups. Specifically, the FP group exhibited a higher mean negative consequences score (4 out of 5) compared to the other groups (3 out of 5). There were no statistically significant differences observed in emotional representation, positive consequences, or positive control scores between the groups.

Regarding self-rated memory, the FI and FP groups reported lower scores, but a majority proportion (67% and 72%, respectively) still rated their memory as at least good. Similarly, for overall self-rated health, the FI and FP groups also showed substantial proportions (64% and 72%, respectively) rating their health as at least good.

## Discussion

In this study, we aimed to explore differences among individuals aged 65 or older living in the community in Ireland, focusing on those classified as frail using three different scales: Fried's Frailty Phenotype (FP), Frailty Index (FI), and Clinical Frailty Scale (CFS).

In terms of measured characteristics, pairwise comparisons demonstrated that, when compared to the non-frail group, the frail groups were older, had more limiting long-term illnesses, experienced more polypharmacy, had higher proportions of falls in the past year, and exhibited worse cognition. Within the frailty groups, the FI group had a majority of women, while in the FP group, men were the majority. As expected, given the explicit inclusion of unexplained weight loss in its criteria, the FP group had the lowest BMI. Also as expected, given the inclusion of several morbidities as constituent deficits, the FI group exhibited the highest multimorbidity. On the other hand, mirroring the importance of disability in the generation of the CFS classification tree, the CFS group concentrated on disability. In that regard, our findings align with the specific domains intended to be assessed by each frailty scale. These findings emphasise the complementary value of using multiple frailty identification scales to gain a more comprehensive understanding of how frailty relates to various geriatric syndromes in older individuals. Indeed, by assessing all geriatric domains, Comprehensive Geriatric Assessment (CGA) provides a more complete picture than that offered by frailty identification tools.

In terms of the lived experience, when compared to the non-frail group, the frail groups exhibited a higher level of fear of falling, more depressive symptoms, lower quality of life, decreased participation in organisations and active leisure, worse perceptions of ageing, lower self-rated health, and poorer self-rated memory. Among the frailty groups, the FI group appeared to have the most adverse profile in terms of quality of life, perceptions of negative consequences of ageing, as well as lower self-rated health and poorer self-rated memory.

While the aforementioned differences indicate the expected fact that measured characteristics and lived experiences are more adverse in frail individuals compared to non-frail ones, the most remarkable finding of our study is that, even among individuals classified as frail, the extent of the negative impact was not as pronounced as one might assume. This observation challenges the traditional perception that frailty invariably leads to very poor quality of life and mental well-being. Indeed, our data indicated that even in the presence of physical limitations and health challenges, a significant proportion of frail individuals reported positive perceptions of their health, mental status, and overall well-being.

Previous research in the literature has consistently associated frailty with negative outcomes, including negative perceptions and social stigma [36, 37]. Shafiq et al.'s [38] recent review further highlighted that frailty is often perceived negatively, linked to the natural ageing process, increased dependency, loss of identity, social exclusion, and stigma. In contrast, our study challenges this prevailing assumption by providing evidence that good lived experiences are prevalent among frail individuals. This timely counterpoint is essential, because, as noted by other scholars, labeling individuals as 'old and frail' may contribute to the development of a frailty identity, leading to attitudinal and behavioural confirmation, which includes a reduced interest in participating in social and physical activities, poor physical health, and increased stigmatisation [39–42]. Our findings emphasise the resilience and adaptability of frail individuals, offering a more nuanced perspective on the lived experience of frailty and questioning the common belief that frailty is not worth living. Indeed, in our study, the mean scores of the evaluated scales indicated low levels of negative mental states, such as worry, anxiety, depression, and loneliness, among the frailty groups. Moreover, perceptions of quality of life and self-perception of ageing, health, and mental status were relatively high.

The relationship between mood disorders and frailty has been the subject of previous research, although the exact mechanisms and direction of this relationship remain not fully understood. Some studies have demonstrated an association between frailty and mood disorders, such as depression and anxiety, particularly in specific diseases like cardiovascular conditions [43] or oncological diseases [44]. These authors suggest that disease-specific symptoms or directed treatments may exacerbate both physiological and psychological status, emphasising the importance of early detection and screening for mood disorders and worry in frail individuals, as they are related to poor clinical outcomes.

Similarly, Ní Mhaoláin et al. [45] found higher levels of anxiety and depression among participants classified as pre-frail or frail according to the FP scale. It is worth noting that the prevalence of frailty in these aforementioned studies was higher compared to our study, with a greater proportion of participants exhibiting positive screening tests for mood disorders. The observed disparity in frailty prevalence between our study and previous research, along with the fact that our sample was community-based rather than clinic-based, may account for the differences in our findings.

In our study, the scores of the UCLA loneliness scale were found to be quite low, and the majority of participants reported having more than two close social ties. Interestingly, participants identified as frail by the FP scale were the ones who exhibited lower levels of formal organisational involvement and participated less in social and solitary activities. These findings may be attributed to the physical limitations detected by this specific scale, which could impact their engagement in social and leisure activities. Alternatively, they may be explained by the higher proportion of men in the FP group, and the possibility that gender-specific social behavioural aspects may be in the mix [46].

A recent scoping review demonstrated a link between loneliness, social isolation, and frailty, although the evidence supporting a clear interplay between these factors remains limited [47]. Moreover, the concept of social frailty has emerged, highlighting the need for a comprehensive evaluation of the social environment. While a consensus on its evaluation is yet to be established, it typically includes aspects such as living arrangements, engagement in outdoor activities, frequency of friends’ visits, feelings of helpfulness, and regular communication with someone each day. Social frailty has been associated with a higher risk of disability [48] and worse health-related quality of life [49], underscoring the importance of considering social factors in the context of frailty and overall well-being.

Our study has several strengths that contribute to its significance. First, it offers a comprehensive evaluation of the lived experiences of individuals with frailty living in the community, making it, to our knowledge, the first of its kind. Second, the inclusion of a large number of participants from TILDA enhances the reliability and relevance of our findings to the broader non-clinical community-dwelling population in Ireland.

Our main aim was to investigate differences in lived experiences between frail and non-frail individuals. However, a secondary aim was to examine differences among various frailty classifications (i.e., FP, FI, and CFS), which required that the frailty groups be mutually exclusive. While including overlapping frailty classifications might have resulted in higher reported frailty prevalences, it would have hindered our ability to discern similarities or differences between the various frailty classifications. Nonetheless, one limitation of our study is the relatively lower prevalence of frailty compared to previous research conducted in clinical settings. For instance, the FP group was notably small, which limited the statistical power of comparisons involving this particular group. This disparity may restrict the external validity when extrapolating our findings to clinical or institutionalised populations. In that regard, it is vital for clinicians not to inappropriately extrapolate their experiences from clinical populations to the broader non-clinical population, because the characteristics and needs of individuals seeking clinical care may differ significantly from those of community-dwelling individuals.

Our study is cross-sectional in nature and given that a frailty state can be of variable duration in individuals and relapse and remit over time, further research with longitudinal data may provide additional insights. Another limitation worth noting is our reliance on self-report for data collection, encompassing both the measured characteristics and the lived experience aspects. While self-report measures are practical and commonly used in population-based surveys, they may be subject to biases and inaccuracies, which could influence the validity and reliability of our results. Despite the possibility of social desirability bias, it is important to highlight that the average MMSE scores were above the cut-off of 24 points [50] for all groups. This suggests that, on average, participants exhibited good cognitive status, which may provide some reassurance regarding the validity and accuracy of the self-reported data collected in our study. However, it is essential to remain cautious and consider potential biases when interpreting the findings. We recognise that the gold standard data modality for analysing lived experiences is qualitative, rather than quantitative. While our study utilised quantitative measures to evaluate various aspects of lived experiences, we acknowledge that qualitative methods, such as interviews and in-depth narratives, provide a more nuanced and in-depth understanding of individuals' experiences. These qualitative approaches can capture the richness and complexity of lived experiences, allowing for a deeper exploration of emotions, perspectives, and contextual factors that quantitative measures may not fully capture.

In conclusion, our study emphasises that beyond identifying frailty, it is crucial to consider additional aspects of health and well-being to comprehend the lived experience of individuals with frailty fully. The relatively low levels of negative mental states among the frailty groups challenge preconceived notions about frailty's impact on overall well-being. These findings underscore the necessity of re-evaluating the perception of frailty among both the general population and healthcare professionals. By understanding the lived experiences of individuals with frailty, we can develop more holistic and person-centred approaches to support their well-being and quality of life. Further research is warranted to explore these aspects in diverse populations and to develop targeted interventions that address the multifaceted nature of frailty.
